# An Extended Hypofractionated Palliative Radiotherapy Regimen for Head and Neck Carcinomas

**DOI:** 10.3389/fonc.2018.00206

**Published:** 2018-06-11

**Authors:** Michael Laursen, Lena Specht, Claus Andrup Kristensen, Anita Gothelf, Mogens Bernsdorf, Ivan Vogelius, Jeppe Friborg

**Affiliations:** Department of Oncology, National University Hospital, Rigshospitalet, Copenhagen, Denmark

**Keywords:** head–neck cancer, palliative treatment, radiotherapy, hypofractionation, IMRT

## Abstract

**Background:**

Palliative radiotherapy to patients with head and neck cancer is often necessary, but there is a substantial variation in the treatment regimens reported in the literature, and consensus on the most appropriate schedules does not exist. In order to minimize acute toxicity while at the same time trying to achieve prolonged tumor control, a long hypofractionated regimen has been used routinely in Denmark. In the current retrospective study, we investigated the outcome in patients intended for palliative radiotherapy with this regimen.

**Materials and methods:**

Patients with newly diagnosed head and neck cancer treated with palliative radiotherapy of 52–56 Gy in 13–14 fractions twice weekly from 2009 to 2014 were included. Patients were excluded if they had previously received radiotherapy. Data on disease location, stage, patient performance status (PS), treatment response, acute skin and mucosal toxicity, and late fibrosis were collected prospectively and supplemented with information from medical records.

**Results:**

77 patients were included in the study. Fifty-eight patients (75%) completed the intended treatment. Loco-regional tumor response (complete or partial) was evaluated 2 months posttreatment and observed in 45% of the entire population corresponding to 71% of patients alive. PS had a significant influence on survival (*p* = 0.007) and on not completing the intended treatment. Grade III or IV acute mucositis were observed in 25%, and grade III or IV acute dermatitis observed in 15%.

**Conclusion:**

Palliative hypofractionated radiotherapy with 52–56 Gy in 13–14 fractions shows good tumor response and tolerability in a vulnerable patient population. However, it may not be suited for patients in poor PS.

## Introduction

Cancer of the head and neck is a common malignant disease. In 2012, there were 686,000 new cases, accounting for approximately 5% of all new cancer cases worldwide (1). This included cancers of the oral cavity, pharynx, and larynx, with most of them being squamous cell carcinomas. Patients with head and neck cancer often present with pain and dysphagia as primary symptoms, and left untreated the prognosis for these patients is very poor with a median survival below 4 months (2).

Curative radiotherapy of head and neck cancer is a time-consuming and intensive treatment, associated with significant morbidity, both in the acute and late setting (3). Acute morbidity occurs over a period of several weeks to months and includes severe mucositis, pain, and dysphagia, which is not tolerable for all patients. However, as advanced head and neck cancer is often associated with severe local morbidity, including ulceration, pain, and dysphagia, disease control and symptomatic treatment are still necessary. Therefore, palliative radiotherapy is a reasonable treatment option in patients with primary metastatic disease or when treatment of locally advanced disease with curative intent is not possible due to comorbidity or poor performance status (PS).

There is no international consensus on the schedule of palliative radiotherapy and many different regimens have been proposed, often based on local tradition and infrastructure settings.

Published regimens include the QUAD-shot consisting of 3.5 Gy twice-daily fractions given over two consecutive days to a total of 14 Gy per cycle, with a 3- to 4-week break between cycles, with a maximum of three cycles (4–10). Other examples include 30 Gy in 10 fractions given over 2 weeks (11–13), 48 Gy in 12 fractions 3–4 times a week (14), 20 Gy in five fractions over five consecutive days (15–17), the “0–7–21” regimen (24 Gy in three fractions of 8 Gy each, given on days 0, 7, and 21) (18), 40 Gy in 10 fractions, twice weekly (19), 50 Gy in 20 fractions split course (12), and 50 Gy in 16 fractions, five fractions/week (20).

Thus, there are significant variations in course duration and maximum doses delivered, and this reflects slightly different agendas, from the aim of obtaining swift palliation with short schedules (e.g., 20 Gy/5 fx or 30 Gy/10 fx) to schedules aimed at prolonged disease control (14, 20). The challenges associated with the latter schedules are the significant acute toxicity which approaches the severity seen in radical treatment. In order to find a compromise between acute toxicity and prolonged disease control in patients not suitable for radical treatment, but where prolonged disease control was desirable, a palliative radiotherapy schedule consisting of 52–56 Gy in 13–14 fractions, two fractions per week has been used in Denmark for head–neck cancer.

In this study, we retrospectively investigated the outcome in patients treated with this schedule between 2009 and 2014 at Copenhagen University Hospital Rigshospitalet, Denmark.

## Patients and Methods

### Patient Selection

Patients diagnosed with primary head and neck carcinoma at Rigshospitalet, Denmark between 2009 and 2014 were eligible and included if they were planned to receive palliative radiotherapy with 52–56 Gy/13–14 fractions, two fractions/week. We also included eight patients that earlier had received surgery with curative intent, but later received palliative radiotherapy after a loco-regional recurrence. Patients were excluded if they did not receive any fractions of palliative radiotherapy, had previously received radiotherapy in the head and neck region or had other active cancers. The decision to provide palliative radiotherapy instead of treatment with curative intent was based on an individual assessment, including PS, presence of metastatic disease or presence of significant comorbidities (for example excessive alcohol abuse or heart conditions).

### Data Collection

Data on diagnosis, treatment, and morbidity were reported prospectively in the structured DAHANCA recording sheets and recorded in the DAHANCA clinical database. During treatment, patients were evaluated weekly for symptoms and signs of radiation-induced mucositis and dermatitis with predefined scores. Mucositis was graded as follows; no reactions (0), erythema (1), patchy mucositis (2), confluent mucositis (3), and ulceration (4). Similarly, dermatitis was graded as; no reaction (0), erythema (1), dry desquamation (2), moist desquamation (3), and ulceration (4).

Furthermore, patients were evaluated at 14 days and 2 months after completed treatment according to the DAHANCA guidelines. The evaluation included physical examination, inspection of the oral cavity, palpation of the cervical lymph nodes, and fiberoptic endoscopy of the pharynx and larynx. Evaluation of the macroscopic tumor response on the neck (primary and nodes) was categorized as complete response (CR), partial response (PR), no change (NC), progressive disease (PD), and not evaluable (NE), if the clinician could not evaluate the response. Imaging was not performed on a routine basis and is not part of the follow-up program defined by DAHANCA for patients treated in the palliative setting.

Patients who were seen beyond the 2-month evaluation, were also evaluated for late fibrosis, although not routinely. Fibrosis was evaluated as; no fibrosis (0), discrete fibrosis (1), moderate fibrosis (2), and severe fibrosis (3).

In case of missing data the relevant patient files were examined to complete the data. Approvals were obtained from the Danish Data Protection Agency (j.nr. RH-2016-368) and the National Board of Health (j.nr. 3-3013-1849/1).

### Radiotherapy Planning

For planning purposes, the gross tumor volume (GTV) was delineated and the clinical target volume created by expanding the GTV by a concentric 1-cm margin modified to exclude air and intact bone. All patients were treated in a thermoplastic mask and an additional margin of 0.4 cm was added for the planning target volume. Elective nodal volumes were not included. Organs at risk included the spinal cord, brainstem, parotid glands, submandibular glands, and optic structures when relevant. The maximal dose to the spinal cord and brain stem were 33.9 and 39.3 Gy (biologically equivalent to 39 and 51 Gy in 2-Gy fractions using an α/β ratio of 2 and delivered over 13 fractions). All patients were treated with intensity-modulated radiotherapy (IMRT).

### Statistical Analysis

Descriptive statistics were used to summarize the patients’ characteristics and Kaplan–Meier survival analysis was used to compare survival in different groups with the log-rank test used for significance testing. Overall survival (OS) was calculated from date of first palliative radiotherapy fraction to date of death or October 31, 2016, whichever came first. Due to the unique personal identifier in Denmark and the mandatory national databases, updated vital status was available for all patients. Variables associated with survival were investigated using a multivariate Cox regression model including age, gender, stage, and PS.

### PubMed Literature Search

The reviewed literature was identified through use of the PubMed search engine as of March 1, 2017. Studies were included if they examined the effect of hypofractionated radiotherapy (fraction size > 2 Gy) given with palliative intent to head and neck cancer patients. Studies were excluded if they primarily investigated radiotherapy with concurrent chemotherapy. To the extent it was possible, information on number of patients included in each study, inclusion and exclusion criteria, radiotherapy dose, and fractionation, along with the corresponding equivalent biological doses, proportion of patients with metastases, rates of treatment-related toxicities, OS and compliance, i.e., how many patients finished the intended treatment, was obtained from the literature. Equivalent doses in 2 Gy fractions (EQD_2_) for late effects in normal tissue were calculated for each of the regimens using the equation:

EQD2,normal=DPhys⋅d+αβ2+αβ.

For tumor tissue, we used a modified equation to account for the extended overall treatment time for the palliative regimens, and the equation was as follows:

EQD2,tumor=DPhys⋅d+αβ2+αβ−δprolif⋅(T−Treference),

α/β ratios of 10 and 3 Gy were used for tumor tissue and late effects in normal tissue, respectively. *D*_Phys_ was the total dose given in gray, *d* was the fraction size in gray. In calculating the EQD_2_ in tumor tissue, we used a δ_prolif_ of 0.6 Gy/day. *T* was overall treatment time in days, and *T*_reference_ was set to be the duration of our own regimen, i.e., 46 days. If the treatment was less than 21 days, we assumed there was no further benefit from the shortened duration, and T was set to 21 days.

Some of the quoted studies have also reported biologically equivalent doses, and these may differ from the doses reported in this paper as a result of our attempt to account for the effect of overall treatment time.

## Results

### Patient Characteristics

From January 2009 to December 2014, 77 patients received primary palliative radiotherapy with 13/14 × 4 Gy, and were included in the analysis. The median age was 73 years (range 47–96) and predominantly male (68.8%) (Table 1). The most common tumor sites were the oropharynx (29.9%), oral cavity (20.8%), larynx (18.2%), and hypopharynx (11.7%), and the most common histopathology was squamous cell carcinoma (93.5%). Of the 77 patients, 18 (23.4%) had metastatic disease from the outset. Forty-one (53%) patients presented with pain from tumor or radiating pain and 27 (35%) with dysphagia.

**Table 1:** Study Population (*n* = 77).

Characteristics	*n*(%)
**Gender**	
Male Female	53 (69) 24 (31)

**Primary tumor site**	
Oral cavity Nasopharynx Oropharynx Hypopharynx Larynx Sinonasal Salivary glands Unknown primary tumor	16 (21) 1 (1) 23 (30) 9 (12) 14 (18) 6 (8) 4 (5) 4 (5)

**Histopathology**	
Squamous cell carcinoma Other types	72 (94) 5 (6)

**Stage (UICC 7th)**	
I II III IVa IVb IVc (metastatic) Unknown	2 (3) 4 (5) 7 (10) 28 (38) 15 (20) 18 (24) 3

Fifty-eight patients (75%) completed the intended treatment with 13 or 14 fractions of 4 Gy. Nineteen (25%) patients discontinued the treatment with a median dose of 20 Gy (range 4–40 Gy) delivered. The subgroup of patients that discontinued treatment had a worse pretreatment PS compared to the patients who completed treatment, with 47 and 10% in PS 3–4, respectively. There was no obvious difference in age or stage of the disease between these two groups.

One patient received chemotherapy before radiotherapy and two received chemotherapy after.

### Treatment Response

Two months after completed treatment, 49 (64%) patients were alive, of which 38 (78%) patients were evaluated for tumor response. Twenty-four patients experienced a complete loco-regional response, representing 31% of the entire population, 49% of patients alive 2 months after treatment, and 63% of the patients evaluated for tumor response. Eleven patients experienced a partial loco-regional response, representing 14, 22, and 29%, respectively. Two patients had NC in tumor or nodal site and one patient experienced PD. The remaining patients were NE at the respective sites. Thus, a response (complete or partial) was observed in 45% of the entire population, 71% of patients alive at 2 months, and 92% of patients evaluated at 2 months.

### Survival

**Figure 1:**
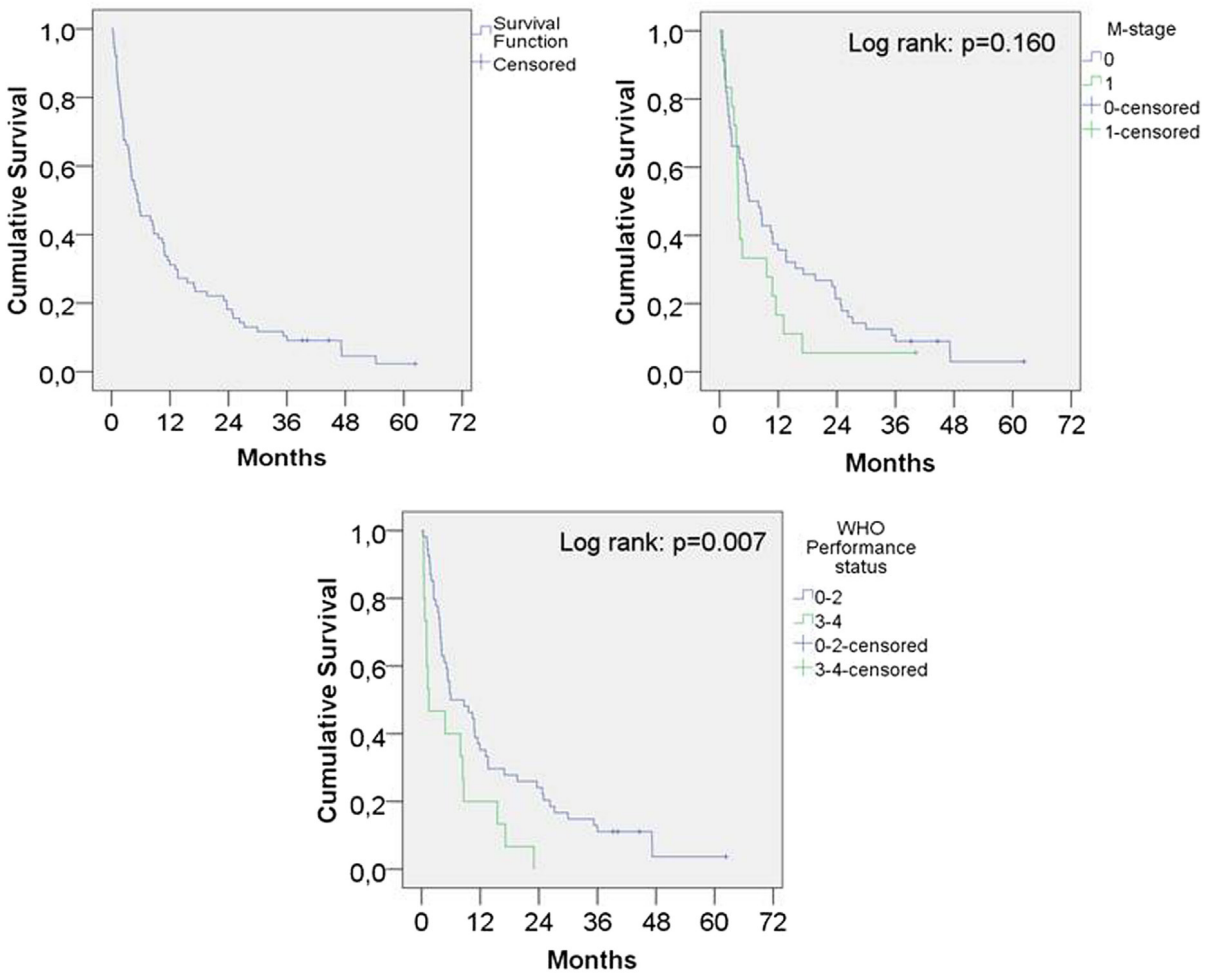
Kaplan–Meier survival function overall and according to presence of metastatic disease and performance status.

The median OS for the entire population was 5.4 months, 1-year survival was 31% and 2-year survival was 18% (Figure 1). The 19 patients who did not complete the intended treatment had a median OS of 1.1 months, compared to 10.4 months survival for patients who completed the treatment (*p* < 0.001).

The median survival for WHO PS 0–2 was 5.9 months and for PS 3–4 1.5 months (*p* = 0.007). PS was not available for eight patients and these patients were not included in the analysis.

Using univariate analysis, PS, but not age, gender, or stage was significantly associated with survival. In the multivariate model containing the same variables, PS maintained the significance and metastatic disease was associated with shorter survival (Table 2).

**Table 2:** Multivariate analysis of prognostic factors for survival in patients receiving palliative radiotherapy.

	*N*	RR	CI	*p*	Trend
**Age**					
<61	14	1			0.10
61–80	38	1.96	0.94–4.11	0.07	
>80	25	1.90	0.80–4.53	0.15	

**Gender**					
Male	53	1			
Female	24	1.02	0.55–1.90	0.94	

**PS**					
0–1	29	1			<0.0001
2	25	1.99	1.06–3.73	0.03	
3–4	15	4.25	1.97–9.14	0.0002	
Missing	8	6.77	2.14–21.41	0.001	

**Stage**					
I–III	13	1			
IVA	28	1.60	0.78–3.26	0.20	
IVB	14	1.26	0.54–2.96	0.59	
Met	18	3.63	1.49–8.86	0.005	

*All variables adjusted for age, gender, performance status (PS), and stage*.

### Toxicity and Late Fibrosis

The radiotherapy was generally well tolerated as seen in Table 3. Of the 57 patients evaluated for acute mucositis, confluent mucositis (grade III) was experienced in 11 (19%) patients and ulceration (grade IV) in 3 (5%) patients. Fifty-four patients were evaluated for acute dermatitis with six (11%) and two (4%) of these experiencing moist desquamation (grade III) and ulceration (grade IV), respectively.

**Table 3:** Highest degree of mucositis and dermatitis registered and highest degree of fibrosis at any time past 2 months posttreatment.

	*n*(%)
**Mucositis (***n*** = 57)**	
No reaction Erythema Patchy mucositis Confluent mucositis Ulceration	4 (7) 17 (30) 22 (39) 11 (19) 3 (5)

**Dermatitis (***n*** = 54)**	
No reaction Erythema Dry desquamation Moist desquamation Ulceration	2 (4) 29 (54) 15 (28) 6 (11) 2 (4)

**Fibrosis (***n*** = 22)**	
None Discrete fibrosis Moderate fibrosis Severe fibrosis	5 (23) 8 (36) 6 (27) 3 (14)

Of the 49 patients surviving more than 2 months after completed treatment, 22 (45%) patients were evaluated at a later follow-up for skin fibrosis of the neck. Slight fibrosis was seen as early as 2 months after completed treatment. The highest recorded degree of fibrosis seen was severe fibrosis in three patients (14%), moderate fibrosis in six patients (27%), slight fibrosis in eight patients (36%), and no fibrosis in five patients (23%).

### Comparable Studies

**Figure 2:**
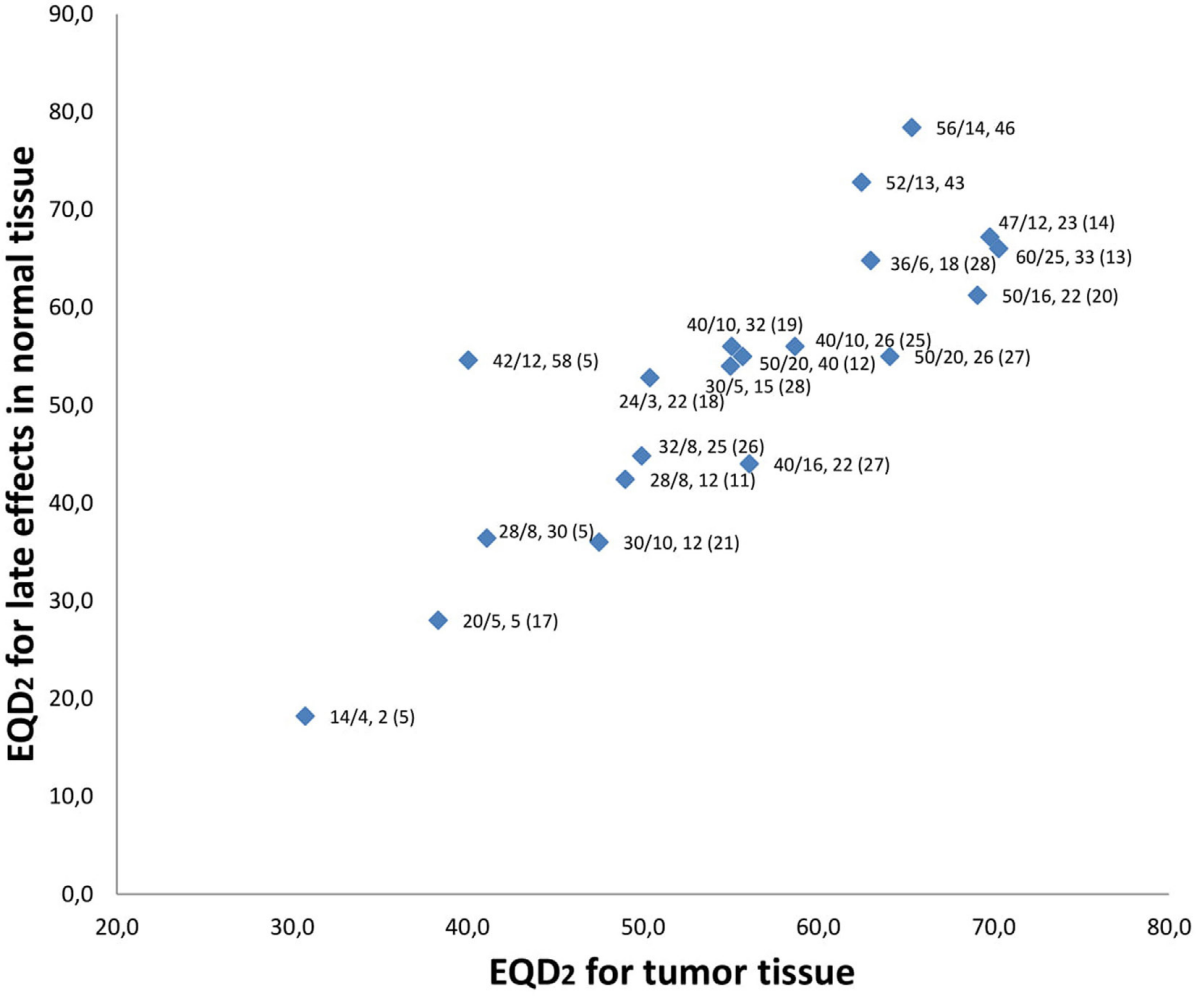
Relation between equivalent doses in 2-Gy fraction (EQD_2_) for late effects in normal tissue and tumor tissue. Data labels are “dose given, (Gy)/number of fractions, overall treatment time (days) (reference).” Current study has no reference and is both shown for 52 Gy in 13 fx and 56 Gy in 14 fx. QUAD-shot represented with doses used by Corry et al. (5). Not all studies represented in Table 4 are referenced as some studies examined the same regimen.

We identified 23 studies that had examined hypofractionated palliative radiotherapy (Table 4). One study (21) was not found through PubMed search. Eleven were retrospective studies (4–7, 9, 12, 14, 18, 22–24) reviewed multiple radiation therapy regimens (4, 11, 12, 22, 23). The studies are shown in Table 4 and the relation between the EQD_2_ for late effects in normal and for tumor tissue is shown in Figure 2. The QUAD-shot-regimen (14 Gy in four fractions given over two consecutive days with at least 6 h apart and can be repeated every four weeks) is illustrated for one, two, and three courses (5) (Figure 2).

**Table 4:** Studies reporting palliative hypofractionated radiotherapy to head–neck cancer patients (only studies with compliance data included).

Reference	*N*	Total dose, Gy	Fx	Duration, days	EQD_2,tumor_, Gy	EQD_2,normal_, Gy (late effects)	Metastases (%)	Median overall survival (months)	Acute toxicity grade 3/4	Compliance (%)
Present study	77	52–56	13–14	43–46	62.5–65.3	72.8–78.4	23	5.4	Muco 24% Derma 15%	75
Finnegan et al. (6)	70	14.8 → 44.4 (QUAD)	4 → 12	2 → 58	30.8 → 40.1	18.2 → 54.6	21	3.85	Muco 9%	76
Murthy et al. (25)	126	32 (→52)	8 (→ 13)	25 (→ 43)	49.9 (→ 62.5)	44.8 (→ 72.8)	0	5.5	Muco 0.7%	74
Lok et al. (7)	75	14.8 → 44.4 (QUAD)	4 → 12	2 → 58	30.8 → 40.1	18.2 → 54.6	55	5.67	Muco 4% Derma 1%	37
Stevens et al. (12)	148	24–70	3–20	40	55.7^a^	55.0^a^	38	5.2	–	70
Ali et al. (21)	30	30	10	12	47.5	36.0	0	–	Muco 0%	100
Pearson et al. (9)	15	14.8 → 44.4 (QUAD)	4 → 12	2 → 58	30.8 → 40.1	18.2 → 54.6	13	4	–	73
Ghoshal et al. (10)	15	14 → 28 (QUAD)	4 → 8	2 → 30	30.8 → 41.1	18.2 → 36.4		–	Muco 0%	87
Agarwal et al. (26)	110	40 (→50)	16 (→ 20)	22 (→ 26)	56.1 (→ 64.1)	44.0 (→ 55.0)	0	–	Derma 14% Muco 66%	78
Chen et al. (4)	60	QUAD; 70 Gy/35 fx; 30 Gy/10 fx; 37.5/15 fx; 20 Gy/5 fx		–	–	–	100	4	Muco 15% Derma 2%	72
Porceddu et al. (27)	35	30 (→36)	5 (→6)	15 (→18)	55.0 (→ 63.0)	54.0 (→ 64.8)	16	6.1	Muco 26% Derma 11% Dysphagia 17%	88
Corry et al. (5)	30	14 → 42 (QUAD)	4 → 12	2 → 58	30.8 → 40.1	18.2 → 54.6	17	5.7	0%	53
Mohanti et al. (17)	505	20	5	5	38.8	28	0	6.6	–	100
Biswal et al. (13)	26	30 (→60)	10 (→ 25)	12 (→33)	47.5 (→ 70.3)	36.0 (→ 66.0)	–	12	–	100

*→, dose escalation to*.

*^a^Calculation made for regimen of 50 Gy in 16 fractions, 5 fx/week, separated by a 2-week break*.

*derma, dermatitis; EQD2, equivalent dose in 2 Gy fractions; Fx, fractions; Muco, mucositis; N, number of patients; QUAD, QUAD-shot*.

*Compliance does not include the dose escalation in parentheses, but does include the QUAD-shot dose escalation*.

## Discussion

In the present study, we have examined the outcome of palliative radiotherapy with 52–56 Gy in 13–14 fractions, given twice weekly, for head–neck cancer. Treatment response rates in the 38 evaluated patients were excellent with 24 patients having CR and 11 patients having PR, while only two and one had NC and PD, respectively. The regimen showed acceptable tolerability, in that only 14 patients (accounting for 25% of the patients evaluated) experienced mucositis grade 3 or more, and eight patients (15% of evaluated) experienced dermatitis grade 3 or more.

However, 25% of patients did not complete the treatment, mainly due to progression of disease or other circumstances such as dementia, poor PS, and transport issues. These patients generally had a worse PS than patients who completed the treatment but otherwise did not differ in age or stage. Therefore, as a result of the short OS and compliance of patients in poor PS (WHO 3–4), we do not believe that the treatment regimen is suitable for this group of patients on a standard basis.

Although many studies have reported outcomes of palliative radiotherapy in head and neck cancers, a direct comparison between the results is difficult. As an example Teckie et al. (22) only included radiotherapy with fraction doses above or equal to 5 Gy and examined sarcomas and melanomas in addition to squamous cell carcinomas, making their study population diverse. Furthermore, patients were excluded if they did not have at least one follow-up visit 30 days after end of treatment. Their results showed a median OS of 7.2 months and limited acute toxicity (1.6% grade III and IV). Nguyen et al. (18) also excluded patients who had no or only one follow-up after treatment resulting in a median OS of 6.2 months. The exclusion of patients not completing the treatment or missing follow-up may bias results and complicate selection of the appropriate treatment.

The challenges comparing studies directly is also evident from the significant variability in how survival and morbidity are recorded across studies. While we used median OS measured from initiation of radiotherapy, other studies have used mean survival (15) or calculated survival from date of diagnosis (24). For morbidity registration, we have prospectively recoded the standard toxicity measures used by the Danish Head and Neck Cancer Group (DAHANCA) database, while other studies have used the RTOG/EORTC toxicity criteria.

An important aspect of palliative care that we could not examine in this study is quality of life (QoL) changes and symptom relief following the radiotherapy treatment. These outcomes have not been routinely registered in our clinic, and could not be reliably deducted from the medical records. However, symptom relief is likely to be associated with tumor response (14) and the combined partial and CR of 35 in 38 evaluated patients, indicates symptom relief in most patients.

The heterogeneity of palliative treatment schedules are illustrated by the significant variation in biologically equivalent doses to both tumor and for late effects in normal tissue. In our schedule, a fairly high EQD_2_ is delivered in the tumor tissue (62.5–65.3 Gy). However, this is accompanied by a high EQD_2_ for late effects in normal tissue (72.8–78.4 Gy) which can explain the significant degree of moderate-severe fibrosis (>40%) observed in patients evaluated after 2 months. This degree of fibrosis may contribute to dysphagia and additional swallowing assessments may be relevant in long-term survivors. A slightly different strategy is exemplified by the schedule proposed and evaluated by Al-mamgani et al. (20) (the “Christie scheme”). Patients undergoing this treatment receive 50 Gy in 16 fractions, 4–5 fractions/week (depending on the patient’s condition) resulting in a high EQD_2_ in tumor tissue (69.1 Gy, if treated with five fractions/week), while the corresponding EQD_2_ for late effects in normal tissue (61.3 Gy) is lower than in our study. The most significant difference between these two regimens is the higher number of fractions per week and shorter treatment duration in the study by Al-mamgani et al. Tissues with a high cell turnover like tumor and mucosa (a high α/β ratio) will be more affected by high EQD_2,tumor_ doses (Figure 2) resulting in higher rates of acute mucositis and dermatitis. For instance, the “Christie scheme” (20) has one of the highest EQD_2,tumor_ in Figure 2 and also the highest rates of confluent mucositis (60%) and grade III dermatitis (45%) reported. These proportions approach the acute side effects seen in curative treatment schedules and a careful selection of candidates for this palliative treatment may be necessary. In comparison, our schedule with a lower EQD_2,tumor_ generated mucositis grade III and IV in 25% of patients and grade III and IV dermatitis in 15% of patients evaluated. In the study by Al-mamgani et al. patients were mostly treated with 2D technique, while all patients in our study received IMRT, a difference that may influence the presence of acute side effects as the risk of confluent mucositis is higher in patients treated with 2D/3D compared to IMRT (14). Thus, our proposed schedule may be relevant in situations where long-term tumor control is desired, but where the acute toxicity observed in other high-dose palliative schedules (14, 20) is a concern.

In our intention-to-treat group of patients, 25% did not complete the treatment. The analysis points to PS being the most important factor influencing the likelihood of completing the treatment, and shorter treatment schedules may be better suited for patients in poor performance. This could have provided palliation and tumor regression faster, but probably with a shorter overall duration. As PS may be difficult to assess, an alternative for patients in poor performance could be a treatment regimen that could be repeated or expanded based on the response and the constitution of the patients, such as the QUAD-shot (5).

Although the present study is retrospective in nature, patients were evaluated and graded prospectively. However, a limitation is the lack of toxicity and treatment response evaluation in all patients. The lack of complete toxicity evaluation may underestimate the acute morbidity associated with the treatment, just as the lack of CR evaluation may overestimate the treatment effect. At 2 months posttreatment, 49 patients were alive, but only 38 (78%) evaluated. If none of the non-evaluated patients alive had response to treatment, the overall 2-month response rates (combined complete and partial) would be 71%.

The main objective of providing non-radical radiotherapy is to alleviate symptoms, improve QoL and achieve disease control. In order to select the most appropriate radiotherapy regimen and to compare the effects of different regimens, randomized controlled trials (RCTs) are much needed. We believe the hypofractionated regimen used in Denmark with 52–56 Gy in 13–14 fractions twice weekly shows good tumor response and tolerability in this patient population, and would be a good choice for comparison in an RCT. However, it may not be suited for patients in poor performance, but relevant for patients not suited for radical treatment for other reasons (e.g., metastatic disease or comorbidity) when disease control is a priority.

## Ethics Statement

This study was carried out in accordance with the recommendations of The Danish National Board of Health and the Danish National Committee on Health Research Ethics. Because of the retrospective nature of this study, and in full agreement with the Danish National Committee on Health Research Ethics, informed consent was not obtained. The data collection was approved by the The Danish National Board of Health (j.nr. 3-3013-1849/1) and the Danish Data Protection Agency (j.nr. RH-2016-368).

## Author Contributions

ML collected and analyzed all the data, participated substantially in preparation of the manuscript, which he has finally approved. LS, CK, AG, MB, and IV contributed to the design, acquisition and analysis of data, and revision of the manuscript, which they have finally approved. JF was responsible for design of the study, collection and analysis of data, and preparation of the manuscript, which he has finally approved.

## Conflict of Interest Statement

The authors declare that the research was conducted in the absence of any commercial or financial relationships that could be construed as a potential conflict of interest.
